# Temozolomide as Second or Third Line Treatment of Patients with Neuroendocrine Carcinomas

**DOI:** 10.1100/2012/170496

**Published:** 2012-08-22

**Authors:** Ingrid H. Olsen, Jens B. Sørensen, Birgitte Federspiel, Andreas Kjaer, Carsten P. Hansen, Ulrich Knigge, Seppo W. Langer

**Affiliations:** ^1^Department of Oncology, Rigshospitalet, University of Copenhagen, 2100 Copenhagen, Denmark; ^2^Department of Gastrointestinal Surgery C, Rigshospitalet, University of Copenhagen, 2100 Copenhagen, Denmark; ^3^Department of Pathology, Rigshospitalet, University of Copenhagen, 2100 Copenhagen, Denmark; ^4^Department of Clinical Physiology, Nuclear Medicine and PET, Rigshospitalet, University of Copenhagen, 2100 Copenhagen, Denmark; ^5^ENETS NET Centre of Excellence, Rigshospitalet, University of Copenhagen, 2100 Copenhagen, Denmark; ^6^Department of Surgery C 2122, Rigshospitalet, Copenhagen University Hospital, Blegdamsvej 9, 2100 Copenhagen, Denmark

## Abstract

*Background*. Knowledge of the clinical efficacy in recurrent neuroendocrine carcinomas is sparse. Treatment with temozolomide alone or in combination with capecitabine and bevacizumab has recently shown promising results. *Patients and Methods*. Analysis of consecutive patients with neuroendocrine carcinomas (Ki-67 proliferation index >20%) and performance status 0–2 treated with temozolomide 200 mg/sqm orally days 1–5 every 28 days after at least one previous platin-containing chemotherapy regimen. *Results*. Twenty-eight eligible patients received a median of 3 courses. Sixteen patients were evaluable for response: Six achieved stable disease and ten progressed. The median survival for the 28 patients was 3.5 months. Survival in patients with tumors of pancreatic origin (*n* = 7) was 7.0 months versus 2.9 months in non-pancreatic origin (*n* = 21). Patients in PS 0-1 (*n* = 22) had a median survival of 4.5 months versus 1.1 months in patients in PS 2 (*n* = 6). Ki-67 index ≥50% was associated with a significantly shorter median survival than Ki-67 index <50% (2.7 months versus 10.9 months). The treatment was well tolerated. *Conclusion*. Temozolomide monotherapy has limited effect in treatment of recurrent neuroendocrine carcinomas. Second line treatment with temozolomide in combination with other compounds should be further investigated in patients in good performance with Ki-67 index <50%.

## 1. Introduction

Neuroendocrine carcinomas (NECs), WHO 2010 Classification [1] Grade 3, (previously classified as poorly differentiated neuroendocrine carcinomas (PDECs)) are high-grade malignant neoplasms and account for around 10% of all GI neuroendocrine neoplasms (NENs) [2]; however, the true number may be higher. The management of NENs is in part guided by histology. NECs with Ki67 >20% generally has an aggressive clinical course, resulting in a median survival of approximately six months without treatment [2]. First line treatment is commonly a combination of etoposide and cisplatin or carboplatin, resulting in a median overall survival of approximately 15 months [3]. There is no consensus regarding further treatment recommendations.

Temozolomide (TMZ) is an orally available alkylating agent with activity in primary brain tumors and metastatic melanoma. TMZ has a mechanism of action that is similar to streptozocin and dacarbazine [4].

The role of second line treatment of NECs with TMZ has been sparsely explored. However, a recent retrospective study of 25 patients indicated that TMZ as monotherapy or in combination with capecitabine (+/− bevacizumab) had a promising result concerning tumor response and PFS [5].

We present a retrospective analysis of treatment efficacy and tolerability in 28 patients with advanced stage NEC (WHO G3) who received TMZ monotherapy as second or third line treatment after first line carboplatin and etoposide at the Department of Oncology, ENETS NET Centre of Excellence, Rigshospitalet, Copenhagen, Denmark.

## 2. Material and Methods

Twenty-eight patients with NEC (Ki-67 > 20%) and disease progression after previous exposure to carboplatin and etoposide, adequate hematological and hepatic function, and WHO performance status 0–2 were eligible for TMZ treatment and further analysis. Informed consent was obtained in all patients.

Patient characteristics and the location of the primary tumor and metastases are given in Table 1.

All patients had measurable disease according to the Response Evaluation Criteria in Solid Tumors (RECIST 1.0). No patients were lost to follow up. All patients had disseminated disease.

The treatment consisted of oral TMZ 200 mg/sqm day 1–5 every 28 days until disease progression or intolerable toxicity. Oral ondansetron 16 mg daily, days 1–5, was given as standard prophylactic antiemetic therapy. Radiological evaluation with CT scanning was performed before and after every three courses. Clinical and biochemical assessment was carried out before every course.

### 2.1. Immunohistochemistry

The Ki-67 index was determined immunohistochemically by applying a monoclonal mouse anti-human Ki-67 antigen (DAKO Clone MIB-1, Dako Denmark A/S, Glostrup, Denmark), Code M7240. Twenty hot spot areas were estimated and the mean percentage of Ki-67 cells calculated. Polyclonal Rabbit Anti-Human Chromogranin A (Dako Denmark A/S, Glostrup, Denmark) Code A 0430 was used for the demonstration of chromogranin in the tumor tissue. Synaptophysin was demonstrated using Monoclonal Mouse Anti-Human Synaptophysin (Clone Snp 88, BioGenex Laboratories Inc., Fremont, U.S.A.), Code MU363.

### 2.2. Statistical Analysis

Survival statistics was performed using Kaplan-Meier curves and log rank tests using the Graph Pad Prism v. 5 software (La Jolla, USA). Qui-square test was used to compare binominal variables between groups of patients when appropriate.

## 3. Results

**Figure 1:**
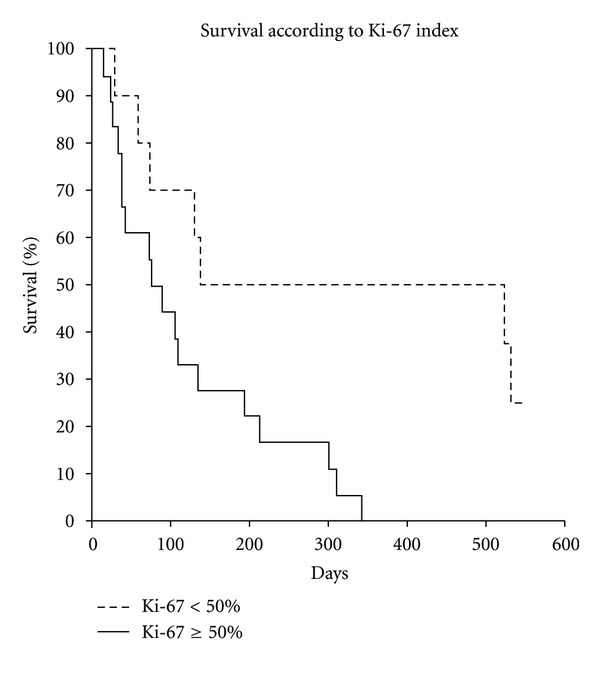
Kaplan-Meier survival curves for patients with Ki-67 index <50% versus ≥50%.

Patients received a median of 3 courses (range 1–12) of treatment. Nineteen patients (68%) received TMZ as second line treatment, and 9 patients (32%) as third line therapy. Twelve patients had no evaluation scans (death, poor condition), leaving 16 patients (67%) eligible for response evaluation No patient achieved complete response (CR) or partial response (PR), six patients (38%) had stable disease (SD), and ten (62%) had progressive disease. Accordingly, the disease control rate (CR + PR + SD) was 38%. The overall median survival time (OS) for the 28 patients was 3.5 months and the overall median progression free survival (PFS) time was 2.4 months. Subgroup analysis showed no difference in median OS between patients who received TMZ as second line chemotherapy (*n* = 19; OS 3.5 months) and in patients who received TMZ as third line therapy (*n* = 9; OS 4.4 months). This difference was not statistically significant (*χ* ^2^ = 0.43, *P* = 0.51). The subgroup that received only one course (*n* = 10) had a shorter median survival of 1.1 months versus 6.6 months in patients who received more than one course (*n* = 18). This difference was highly significant (*χ* ^2^ = 23.70, *P* < 0.0001). The seven patients with pancreatic NEC had a median OS of 7.0 months versus 2.9 months in patients with nonpancreatic NEC (*n* = 21) (*χ* ^2^ = 0.16, *P* = 0.69). The PFS was 3.3 months versus 1.9 months, respectively (*χ* ^2^ = 1.71, *P* = 0.19). Patients in WHO PS 0-1 (*n* = 22) had a median survival of 4.5 months versus 1.1 months in patients in WHO PS 2 (*χ* ^2^ = 15.55, *P* < 0.0001). The patients in WHO PS 0-1 received a median of 3 courses of TMZ (range 1–12) versus 1 course (1–3) in PS 2 patients. Patients with a Ki-67 index ≥50% had a significantly shorter median survival of 2.7 months compared to 10.9 months in patients with Ki-67 index <50%, (*χ* ^2^ = 7.08, *P* < 0.0001) (Figure 1). There were no statistical differences when comparing survival times in CgA positive versus CgA negative patients, and CgA positive versus CgA negative/weakly positive patients, respectively. Comparing SRS positive versus SRS negative patients and patients with positive SRS (>liver uptake) versus SRS < liver uptake, respectively, showed no statistical significant influence of SRS status on survival. There were no treatment-related deaths. One patient (4%) experienced grade 3 leucopenia and two patients (7%) had grade 4 thrombocytopenia. None of the patients had febrile neutropenia. Emesis was well controlled on the standard prophylactic antiemetic therapy.

## 4. Discussion

The specific aims of our retrospective study were to elucidate and evaluate the efficacy and tolerability of monotherapy with TMZ as second and third line treatment after platin-based chemotherapy in patients with NECs (WHO G3). Usually these patients have a very poor prognosis with a short-term survival. In our material, none of the 28 patients had objective response (CR + PR) to treatment, but a disease control rate of 38%. The OS and PFS after start of TMZ treatment were 3.5 months and 2.4 months, respectively. The first report on second and third line TMZ-based chemotherapy in 25 patients with NECs after progression on first line chemotherapy (cisplatin/etoposide or docetaxel and doxorubicin) [5] showed overall response rate of 33% and disease stabilization in 38% of the patients. The median PFS was 6 months and the median OS was 22 months. The patients were treated with TMZ alone (*n* = 5) or in combination with capecitabine (*n* = 19) of which a subgroup also had bevacizumab (*n* = 7). Adding capecitabine and bevacizumab to TMZ did not seem to have any additional effect. However, the number of patients in each group was small [5], which may hide a beneficial effect of combined therapy. These data are indicative of a promising effect of TMZ in NECs; however, data may not be comparable to our study due to differences in the selection of patients. We found a median Ki-67 proliferation index of 50% in our patients, whereas half of the cohort in the other study [5] had a Ki-67 at 20–30%, which may account for the difference in the present results. Although not obvious in the recent study [5], we had a high inclusion (21%) of patients with PS 2, which may affect the results.

TMZ and capecitabine-based regimes are associated with relatively high tumor response rates in patients with well or moderately differentiated pancreatic NENs (G1 and G2) [4, 6, 7]. We showed a trend towards longer median overall survival for patients with primary pancreatic NECs (7.0 months) versus patients with nonpancreatic NECs (2.9 months) after receiving TMZ as monotherapy. The PFS was 3.3 months versus 1.9 months, respectively. Although different NEN populations, the results are supported by a phase 2 study of 29 patients (28 well-differentiated neuroendocrine tumors and one poorly differentiated neuroendocrine carcinoma) treated with TMZ and thalidomide [8]. The report demonstrated radiologic response rate of 25%—therapy appeared to be most active among metastatic pancreatic NENs with a response rate of 45% [8].

The use of TMZ with capecitabine as first line therapy for pancreatic NENs G1 + G2 has been reported with a response rate of 70% [6]. If these data are consistent and confirmed, TMZ-based chemotherapy regimens with relatively mild toxicity may develop into a future second line or even first line option in these patients.

The role of the nuclear antigen Ki-67 as a prognostic indicator and a surrogate marker for a therapeutic response is still a matter of debate. A review from 2008 [9] endorses Ki-67 immunostaining, particularly in pancreatic NENs with reference to the WHO classification that supports Ki-67 immunostaining as a routine in the immunohistochemical examination of NENs. Our subgroup analysis showed a significantly shorter median survival in patients with a Ki-67 index ≥50% (Figure 1). The study of TMZ-based chemotherapy of NECs [5] also found more responders in patients with Ki-67 <60% than in those with higher Ki-67. This may reflect the difference in tumor biology between tumors with high and low Ki-67, respectively, and thus call for an extended division into subgroups than the current WHO 2010 classification. In contrast to the previous study [5], positive or negative CgA immunohistochemistry or positive or negative SRS were not predictive factors for survival, which may be due to the small sample size.

O^6^-methylguanine DNA methyltransferase (MGMT) is a DNA repair enzyme that is believed to induce cancer cell resistance to O^6^-alkylating agents, for example, TMZ [10–12]. This is, however, not a consistent finding, as other studies found no significant difference in the response rate after TMZ-based regimens when correlated with the MGMT expression [5, 13]. The role of MGMT promoter methylation status as a predictive factor when it comes to NEC patients treated with TMZ is not consistent and was not a part of our retrospective analysis. To look further into the hypothesis of MGMT expression and TMZ response, a study of a greater cohort than ours is needed.

In conclusion, TMZ is an option for palliative treatment in patients with NECs as the toxicity is acceptable, and treatment may be received on an outpatient basis. Overall TMZ had limited effect in the second and third line treatment of patients with NECs in the present study. However, our data indicate that a subset of patients with pancreatic NECs may benefit from the treatment. Further and larger studies of TMZ as a second line treatment of NEC are warranted, particularly in pancreatic NECs and probably in the form of combination chemotherapy. In order to optimize the result of the investigation, it is presupposed that the patients are very carefully selected having high performance status and low Ki-67 indices.

## Figures and Tables

**Table 1:** Patient characteristics

No of patients	28	
Male	16	57%
Female	12	43%
Median age, y, (range)	58	(32–81)
Performance status		
0-1	22	79%
2	6	21%
Primary tumor		
Pancreas	7	25%
Esophagus	3	11%
Gastric	3	11%
Colon	4	14%
CUP (unknown primary)	6	21%
Other sites		18%
Rectum	1	
Prostate	1	
Lung	1	
Kidney	1	
Chromogranin A immunohistochemistry		
Strongly positive	19	
Weak	6	
Negative	3	
Synaptophysin immunohistochemistry		
Strongly positive	28	
Negative	0	
Somatostatin receptor scintigraphy		
Strongly positive (>liver uptake)	8	
Weakly positive (≤liver uptake)	3	
Negative	10	
Not done	7	
Ki-67		
Median, %, (range)	50	(20–100)
≥50%	17	
<50%	11	
Site of metastases		
Liver	24	
Lymph nodes	22	
Lung	4	
Bone	3	
Pelvic, brain, renal, breast, skin, peritoneal	10	
No. of metastatic sites		
1	4	
2	13	
≥3	11	
